# Dual-Stage AI Model for Enhanced CT Imaging: Precision Segmentation of Kidney and Tumors

**DOI:** 10.3390/tomography11010003

**Published:** 2025-01-03

**Authors:** Nalan Karunanayake, Lin Lu, Hao Yang, Pengfei Geng, Oguz Akin, Helena Furberg, Lawrence H. Schwartz, Binsheng Zhao

**Affiliations:** 1 Department of Radiology, Memorial Sloan Kettering Cancer Center, New York, NY 10065, USA; lul1@mskcc.org (L.L.); yangh6@mskcc.org (H.Y.); gengp@mskcc.org (P.G.); akino@mskcc.org (O.A.); schwartzl@mskcc.org (L.H.S.); zhaob1@mskcc.org (B.Z.); 2 Department of Epidemiology and Biostatistics, Memorial Sloan Kettering Cancer Center, New York, NY 10017, USA; fuberga@mskcc.org

**Keywords:** kidney cancer, tumor detection and segmentation, deep learning, CECT, vision transformers, convolutional neural networks

## Abstract

Objectives: Accurate kidney and tumor segmentation of computed tomography (CT) scans is vital for diagnosis and treatment, but manual methods are time-consuming and inconsistent, highlighting the value of AI automation. This study develops a fully automated AI model using vision transformers (ViTs) and convolutional neural networks (CNNs) to detect and segment kidneys and kidney tumors in Contrast-Enhanced (CECT) scans, with a focus on improving sensitivity for small, indistinct tumors. Methods: The segmentation framework employs a ViT-based model for the kidney organ, followed by a 3D UNet model with enhanced connections and attention mechanisms for tumor detection and segmentation. Two CECT datasets were used: a public dataset (KiTS23: 489 scans) and a private institutional dataset (Private: 592 scans). The AI model was trained on 389 public scans, with validation performed on the remaining 100 scans and external validation performed on all 592 private scans. Tumors were categorized by TNM staging as small (≤4 cm) (KiTS23: 54%, Private: 41%), medium (>4 cm to ≤7 cm) (KiTS23: 24%, Private: 35%), and large (>7 cm) (KiTS23: 22%, Private: 24%) for detailed evaluation. Results: Kidney and kidney tumor segmentations were evaluated against manual annotations as the reference standard. The model achieved a Dice score of 0.97 ± 0.02 for kidney organ segmentation. For tumor detection and segmentation on the KiTS23 dataset, the sensitivities and average false-positive rates per patient were as follows: 0.90 and 0.23 for small tumors, 1.0 and 0.08 for medium tumors, and 0.96 and 0.04 for large tumors. The corresponding Dice scores were 0.84 ± 0.11, 0.89 ± 0.07, and 0.91 ± 0.06, respectively. External validation on the private data confirmed the model’s effectiveness, achieving the following sensitivities and average false-positive rates per patient: 0.89 and 0.15 for small tumors, 0.99 and 0.03 for medium tumors, and 1.0 and 0.01 for large tumors. The corresponding Dice scores were 0.84 ± 0.08, 0.89 ± 0.08, and 0.92 ± 0.06. Conclusions: The proposed model demonstrates consistent and robust performance in segmenting kidneys and kidney tumors of various sizes, with effective generalization to unseen data. This underscores the model’s significant potential for clinical integration, offering enhanced diagnostic precision and reliability in radiological assessments.

## 1. Introduction

Kidney cancer is among the most frequently diagnosed cancers worldwide, and early detection is essential for improving survival rates. Renal cell carcinoma (RCC) is the predominant type of kidney cancer, and its clear cell variant (ccRCC) accounts for approximately 85% of all RCC cases, making it the most malignant subtype [1]. The aggressive nature of ccRCC is further evidenced by its potential to metastasize to other sites, including the lungs, liver, bones, and lymph nodes [2,3]. Computed tomography, a frequently used imaging modality for screening ccRCC, benefits from CECT to improve the visualization of renal tumors and vascular structures [4]. However, accurately identifying ccRCC in CECT images remains challenging, even for experienced radiologists, due to overlapping image features [5] and the presence of benign tumors like angiomyolipoma and oncocytoma [6,7], which have similar imaging characteristics [8]. This is particularly challenging for detecting and characterizing T1a-stage tumors (≤4 cm) that are confined within the kidney [9,10].

An automated AI pipeline enhances kidney cancer diagnosis by integrating imaging, radiomics, genomic, and clinical data through deep learning (DL) for kidney and kidney tumor detection, segmentation, and characterization. This study focuses on the initial pipeline stages: detecting and segmenting kidneys and kidney tumors. Accurate, reproducible segmentation is crucial for diagnosis, treatment planning, and monitoring. Manual segmentation by radiologists is tedious, time-consuming, and variable, leading to inconsistent outcomes, particularly when tumors are indistinguishable radiologically and macroscopically without pathology [11,12].

Despite advances in DL for medical imaging, kidney tumor detection and segmentation still face challenges, particularly with small tumors. These tumors often lack distinctive features and exhibit low soft-tissue contrast in the background of normal kidney parenchyma, especially in early stages, leading to decreased sensitivity and increased false positives (FPs) in state-of-the-art (SOTA) models [13,14]. However, early detection is crucial, as identifying tumors when they are smaller than 1 cm significantly increases the likelihood of successful treatment with less invasive interventions [15]. By improving the sensitivity of DL models in detecting and segmenting small tumors, we can better identify those more likely to be cured before they reach a size where the risk of metastasis and complexity of treatment increase.

Recently, CNNs and ViTs have shown promising results in medical imaging [16,17,18]. CNNs are effective at feature extraction and local information integration [18,19,20] but struggle with global spatial context [21], potentially reducing segmentation performance for large organs. ViTs overcome this limitation by capturing long-range dependencies, although they are less effective with low-level features [22,23]. While ViTs excel at segmenting large organs, they are less accurate for small tumors [24]. Moreover, ViTs require significantly larger datasets and greater computational resources for training compared to CNNs due to their reliance on self-attention mechanisms and absence of inductive biases.

In 2015, UNet [25], a U-shaped CNN, set the benchmark for medical image segmentation, later leading to nnUNet [26]. nnUNet excelled in kidney and kidney tumor segmentation, winning the KiTS Grand Challenge (Kidney Tumor Segmentation Challenge) in 2019 [27] and 2021 [28]. Approaches to kidney tumor segmentation models can be categorized as single-stage (SS) and dual-stage (DS). DS models, similar to a coarse-to-fine strategy, use two networks: one for initial localization and another for refined segmentation. In contrast, SS models use a single network to simultaneously segment the kidney organ and tumors. A CNN-based DS model [29] uses AlexNet [30] to select kidney slices along the Z-axis, followed by 2D UNet segmentation, trained solely on the KiTS19 dataset (210 CECT scans), which may limit generalizability. Another DS model [31] utilizes a 2D UNet for both kidney and kidney tumor segmentation, training on KiTS19 and a privately-owned dataset (315 RCC CECT scans), unlike our use of only public data. A study [32] employed a pretrained 3D UNet for initial masks and another for fine segmentation, validated on KiTS19 and a small external set (20 patients), limiting robust generalization. Another model [33] uses a low-resolution 3D UNet for region-of-interest extraction, followed by a finer 3D UNet, tested on a privately owned dataset (441 CECT scans).

As CNNs laid the foundation for kidney and kidney tumor segmentation, recent advances have incorporated ViTs to address some of their limitations. The study reported in [34] proposed a modified UNETR [35] for kidney and kidney tumor segmentation in CECT scans, replacing multi-head self-attention with squeeze and excitation layers for efficiency. Another approach [36] combines convolution and transformer layers for multi-scale feature learning. Using 2D slices may not fully capture the 3D spatial information of CECT scans. A hybrid CNN and ViT model proposed in [37] targets kidney and kidney tumor segmentation, but the authors note limitations in generalization and robustness. While integrating transformers with CNNs, such as in the UNETR model, can enhance performance, it also introduces design and training complexities, including potential increases in FPs for smaller tumors. In [38], a transformer–convolution hybrid network was proposed for kidney segmentation using minimal labeled data, inspiring our method that employs a ViT for kidney segmentation in the first stage.

In this paper, we aim to enhance the nnUNet 3D network using a DS approach. First, we segment the kidney using SwinUNETR [39], a ViT-based network that combines the strengths of Swin transformers with the U-Net architecture. By leveraging hierarchical feature extraction and self-attention mechanisms, SwinUNETR effectively captures global context and spatial details, making it particularly suitable for segmenting large organs. Then, we enhance nnUNet 3D for tumor detection and segmentation by incorporating residual blocks (RBs) and an attention mechanism, inspired by ResNet [40], to capture complex features and improve precision. Our goal is to develop a robust AI model that increases detection sensitivity, reduces FPs, and improves segmentation accuracy for kidney tumors.

## 2. Materials and Methods

This section outlines the dataset and model development.

### 2.1. Dataset Information

We used the public KiTS23 dataset for training and internal validation, following an 80/20 split: 389 CECT scans for training and 100 scans for internal validation. The private dataset from our institute, consisting of 592 CECT scans, was used for model validation. The KiTS23 dataset includes corticomedullary and nephrogenic phase scans, with slice thicknesses ranging from 0.5 mm to 5.0 mm. The training set contains various tumor subtypes: ccRCC with 247 cases (63.49%), papillary RCC with 39 cases (10.03%), chromophobe RCC with 33 cases (8.48%), oncocytoma with 19 cases (4.88%), transitional cell carcinoma with 17 cases (4.37%), and other subtypes with 34 cases (8.74%). The internal validation set comprises 100 cases, with subtype proportions of 63% ccRCC, 6% papillary RCC, 5% chromophobe RCC, 7% oncocytoma, and 19% other subtypes. The private dataset consists exclusively of nephrogenic phase scans with slice thicknesses ranging from 1.0 mm to 7.5 mm, focusing solely on the ccRCC subtype.

### 2.2. Data Preprocessing and Augmentation

Data preprocessing is crucial for effective building of DL models in medical imaging, as it eliminates artifacts, standardizes data, and enhances training efficiency [41]. In our preprocessing steps, we retained only the intensity range between the lower and upper 0.5% of Hounsfield unit values, resampled the data isotropically to a 1.0 mm voxel spacing, and normalized the intensity values by clipping them within this range. Each image was then adjusted to have a zero mean and unit variance based on the dataset’s overall statistics.

Beyond preprocessing, data augmentation is also essential in DL training for medical imaging to enhance target representation diversity. To address the under-representation of small tumors and rare cases, we employed techniques such as random rotations, scaling, mirroring, axis transposition, and gamma corrections to increase variability during training. These augmentations enhanced the model’s robustness and generalization, yielding a 5.5% increase in overall Dice scores during training. Additionally, the augmentation techniques mitigated overfitting and improved balance across various tumor size classes, as evidenced by more consistent performance on the private dataset.

### 2.3. DL Model Development

**Figure 1:**
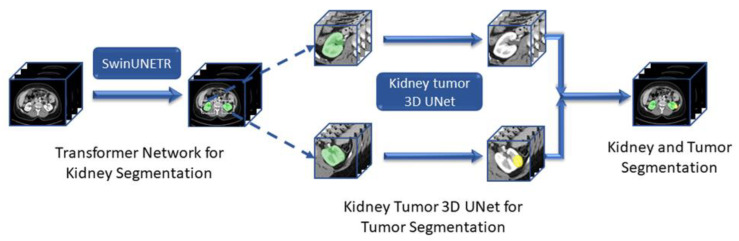
Proposed dual-stage kidney and tumor segmentation framework.

Figure 1 illustrates the proposed DS pipeline for kidney organ and tumor segmentation.

#### 2.3.1. Kidney Organ Segmentation

For kidney segmentation, we used SwinUNETR, chosen for its superior boundary capture and consistency. SwinUNETR uses a shifted window transformer to create non-overlapping 128*3-sized patches from the input 3D data for self-attention computation. Its encoder has four steps, each with two transformer blocks. Encoded features are fed into a CNN-based decoder via skip connections. At each step, features are reshaped and processed through residual blocks with 3D convolutions and instance normalization. Feature map resolution is doubled using deconvolution, and final segmentation outputs are generated with a pointwise convolution and a sigmoid activation function.

We optimized the model with the AdamW optimizer, using an initial learning rate of $3.5\times 10^{-4}$; adaptive weight decay; and DiceCE loss, which combines the Dice and cross-entropy loss functions. We also leveraged transfer learning by initializing the model with pre-trained weights from [42].

#### 2.3.2. Kidney Tumor Segmentation

**Figure 2:**
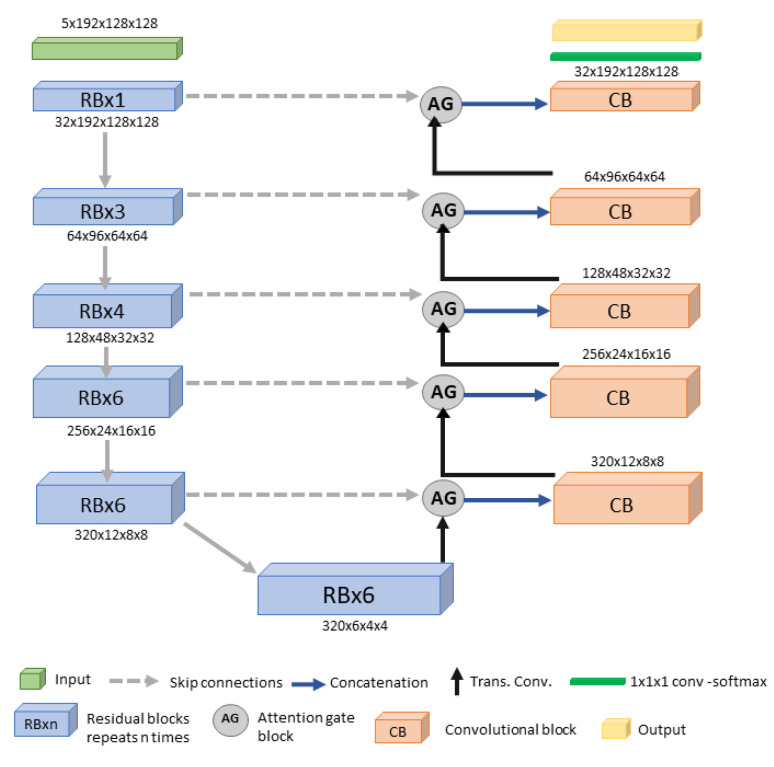
Proposed Kidney Tumor 3D UNet network architecture.

The proposed Kidney Tumor 3D UNet model enhances feature extraction and segmentation accuracy. Configured with a batch size of 5 and a patch size of 192 × 128 × 128, the network (see Figure 2) is optimized for efficient training and precise feature extraction across six stages, with feature map sizes of 32, 64, 128, 256, 320, and 320.

The encoder consists of six stages, each with RBs, which use skip connections to address the vanishing gradient problem and enhance feature learning [27,43]. These blocks, with 1, 3, 4, 6, 6, and 6 RBs per stage, gradually increase feature complexity to capture both low- and high-level features. The decoder mirrors the encoder, focusing on up-sampling to reconstruct high-resolution segmentation maps, using one convolution per stage. LeakyReLU handles non-linearity. Attention gates (AGs) emphasize informative regions by weighting input feature maps, using a sigmoid activation on a linear transformation, followed by element-wise multiplication, to focus on significant areas, improving small and indistinct tumor segmentation and reducing FPs. The model uses a learning rate of 0.001, optimized with stochastic gradient descent (SGD) and DiceCE loss. Both the networks were implemented using Python 3.10 and PyTorch 2.2, with additional dependencies including CUDA 11.1. All training was carried out on an NVIDIA A40 48GB GPU.

### 2.4. Evaliation Metrics

We assessed kidney tumor detection and segmentation using traditional evaluation metrics. For tumor detection, we used sensitivity and the average false-positive count (FPC) per patient. Sensitivity was calculated as the proportion of correctly detected tumors, categorized by size. A 3D object-wise Intersection over Union (IoU) threshold of 0.25 was used to determine detection.

For segmentation, we evaluated using following metrics:

The Dice coefficient (Dice) measures the overlap between the predicted segmentation and the ground truth (GT).

Dice = 2TP/(2TP + FP + FN),(1)

Precision indicates the proportion of true-positive segmentations out of all positive segmentations predicted by the model.

Precision = TP/(TP + FP),(2)

Volumetric Distance (VD) [44] evaluates the difference in volume between the predicted segmentation and the GT, providing insight into the spatial accuracy of the segmentation.

VD = |FN − FP|/(2TP + FP + FN),(3)

The Over-Segmentation Ratio (OSR) evaluates the excess voxels included by the DL model outside the GT region. The OSR is calculated as the number of false-positive voxels divided by the total number of positive voxels in the GT.

OSR = FP/(TP + FN),(4)

The Under-Segmentation Ratio (USR) measures voxels missed by the DL model within the GT region. The USR is calculated as the number of false-negative voxels divided by the total number of positive voxels in the GT.

USR = FN/(TP + FN),(5)

where TP is true positives, FP is false positives, and FN is false negatives.

## 3. Results

The following section presents the detection and segmentation results for kidneys and kidney tumors.

### 3.1. Organ Segmentation

Kidney organ segmentation was evaluated exclusively using KiTS23, as the private data only include kidney tumor annotations. Table 1 shows the quantitative segmentation results for kidney segmentation in KiTS23. The table includes segmentation metrics for the nnUNet-3D and SwinUNETR models, along with the *p*-value for the Dice coefficient.

The results show that SwinUNETR achieves a higher precision (0.97) and Dice (0.97) compared to nnUNet-3D (precision: 0.95; Dice: 0.95), with a statistically significant *p*-value of 0.0498. The lower standard deviations associated with SwinUNETR further highlight its consistency across the dataset. This superior accuracy in segmenting large 3D objects, such as organs, can be attributed to the transformer architecture’s ability to leverage spatial awareness for better distinction between object and background. Consequently, SwinUNETR was selected for stage one of our study to effectively capture kidney boundaries.

### 3.2. Tumor Detection and Segmentation

To evaluate the kidney tumor detection and segmentation, we compare four segmentation models: SS models SS_SwinUNETR and SS_nnUNet-3D and DS models DS_SwinUNETR and DS_nnUNet-3D. For the DS models, the first stage uses the same network (e.g., SwinUNETR for DS_SwinUNETR) to perform organ segmentation before tumor segmentation in the second stage. All models were trained using their provided standard training parameters to ensure a fair comparison. Table 2 presents the sensitivity and FPC values for small, medium, and large tumors for all models, representing the tumor detection results. For small tumors, the proposed Kidney Tumor 3D UNet achieves a sensitivity of 0.90 for KiTS23 and 0.89 for the private data. Benchmark models demonstrate notable improvements with DS configurations. The sensitivity of SS_SwinUNETR for small tumors in KiTS23 increases from 0.62 to 0.67, an 8.06% improvement, when upgraded to DS_SwinUNETR. Similarly, in the private data, sensitivity improves from 0.70 to 0.80, indicating a 14.29% increase. This trend is also observed in nnUNet-3D, where sensitivity for KiTS23 rises from 0.59 to 0.74, a 25.42% improvement from SS to DS and, in the private data, from 0.62 to 0.84, showing a 35.48% improvement.

For medium-sized tumors, all models exhibit high sensitivity. DS_SwinUNETR and Kidney Tumor 3D UNet demonstrated maximum sensitivity for KiTS23, with Kidney Tumor 3D UNet also achieving the highest sensitivity in the private data, showing strong adaptability to unseen data. For large tumors, all models show a variation in sensitivity between KiTS23 and the private data. In KiTS23, SS_SwinUNETR achieves a sensitivity of 0.87, while SS_nnUNet-3D shows an improvement, with a sensitivity of 0.91. DS_SwinUNETR and DS_nnUNet-3D further increase sensitivity to 0.91 and 0.96, respectively. The proposed Kidney Tumor 3D UNet achieves the highest sensitivity of 0.96, representing a 9.3% increase compared to SS_SwinUNETR. In the private data, all models demonstrate excellent performance, with the Kidney Tumor 3D UNet achieving the highest sensitivity of 1.00, alongside DS_SwinUNETR and DS_nnUNet-3D.

Figures S1 and S2 (see the Supplementary Materials) illustrate the sensitivity and FPC across all models for the validation datasets. The Kidney Tumor 3D UNet achieves the lowest FPC in the small tumor category, with 0.23 in KiTS23 and 0.15 in the private data, as well as in the medium and large tumor categories. The figures show a clear improvement in performance from the SS to DS model.

**Figure 3:**
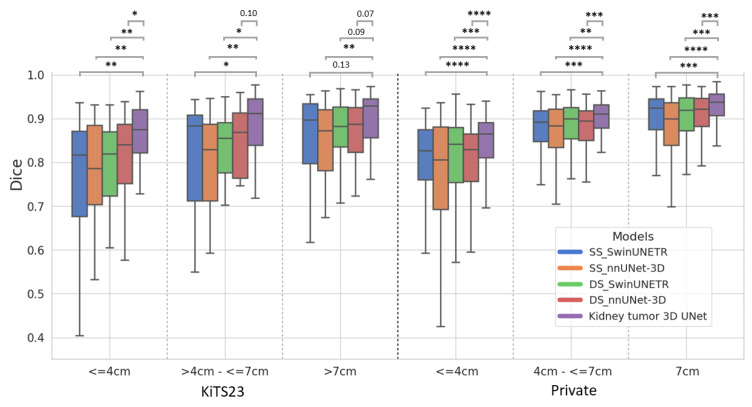
Boxplot of Dice scores with statistical significance for all models across different tumor sizes in KiTS23 and private data. The statistical significance is indicated as follows: **** (*p* ≤ 0.0001), *** (*p* ≤ 0.001), ** (*p* ≤ 0.01), * (*p* ≤ 0.05).

Figure 3 presents boxplots of Dice scores for all models across different tumor sizes in both datasets, accompanied by *p*-values for statistical comparisons. The boxplots clearly illustrate the distribution, median, interquartile range, and outliers, providing a detailed view of the models’ performance. The Kidney Tumor 3D UNet consistently achieves the highest median Dice scores across all tumor size categories, particularly excelling in the segmentation of small tumors. This indicates its ability to handle challenging cases with greater precision. The narrow interquartile range for this model further demonstrates its stability and reliability. The *p*-values confirm statistically significant improvements over other SOTA models, especially for small tumors, where detection and precise segmentation are critical. These findings underscore the advantages of the 3D UNet in achieving robust and accurate segmentation across tumor sizes, with particular emphasis on small, clinically significant lesions.

Table S1 in the Supplementary Materials presents segmentation results for small tumors. The Kidney Tumor 3D UNet outperforms all models, achieving the highest Dice score of 0.84 for both KiTS23 and the private data, indicating excellent overlap with the GT. The VD is lowest for this model, with 0.10 for KiTS23 and 0.11 for the private data, indicating minimal discrepancies between predicted and GT tumor volumes. Comparing these results with DS models, the proposed model shows significant advantages. The Dice scores are 3.7% and 5% higher than DS_SwinUNETR for KiTS23 and the private data, respectively, and 5% higher than DS_nnUNet-3D for both datasets. The Kidney Tumor 3D UNet achieves a lower OSR and USR, with an OSR of 0.12 in KiTS23 and 0.23 in the private data and a USR of 0.18 in KiTS23 and 0.10 in the private data, indicating fewer excess and missed voxels compared to the benchmark models.

Table S2 presents the segmentation results for medium-sized tumors, with the Kidney Tumor 3D UNet achieving the highest Dice scores of 0.89 for both datasets. The model maintains high precision, scoring 0.90 for KiTS23 and 0.86 for the private data. The VD remains low, at 0.06 for KiTS23 and 0.07 for the private data. Additionally, the model achieves OSR values of 0.11 in KiTS23 and 0.17 in the private data and USR values of 0.09 in KiTS23 and 0.07 in the private data. Compared to the results for small tumors in Table S1, the Kidney Tumor 3D UNet demonstrates a reduction in OSR by 8.33% in KiTS23, decreasing from 0.12 to 0.11, and by 26.09% in the private data, decreasing from 0.23 to 0.17. Similarly, USR decreases by 50% in KiTS23, from 0.18 to 0.09, and by 30% in the private data, from 0.10 to 0.07. The average Dice scores for DS models show slight improvements over their SS counterparts. DS_SwinUNETR achieves an average Dice score of 0.87 in both datasets, compared to 0.85 in KiTS23 and 0.86 in the private data for SS_SwinUNETR, representing a 2.4% and 1.2% increase, respectively. Similarly, DS_nnUNet-3D improves to a sensitivity of 0.86 in KiTS23 and 0.87 in the private data, up from 0.83 in KiTS23, which is a 3.61% increase, and from 0.85 in the private data, which is a 2.35% increase for SS_nnUNet-3D. Table S3 presents the segmentation performance for large tumors in KiTS23 and the private data. The Kidney Tumor 3D UNet excels, achieving the highest Dice scores of 0.90 in KiTS23 and 0.92 in the private data. It also demonstrates high precision, with scores of 0.93 in KiTS23 and 0.90 in the private data. The model maintains low VD values of 0.06 in KiTS23 and 0.04 in the private data, low OSR values of 0.07 in KiTS23 and 0.12 in the private data, and low USR values of 0.11 in KiTS23 and 0.05 in the private data, demonstrating robust performance, even for larger tumors.

**Figure 4:**
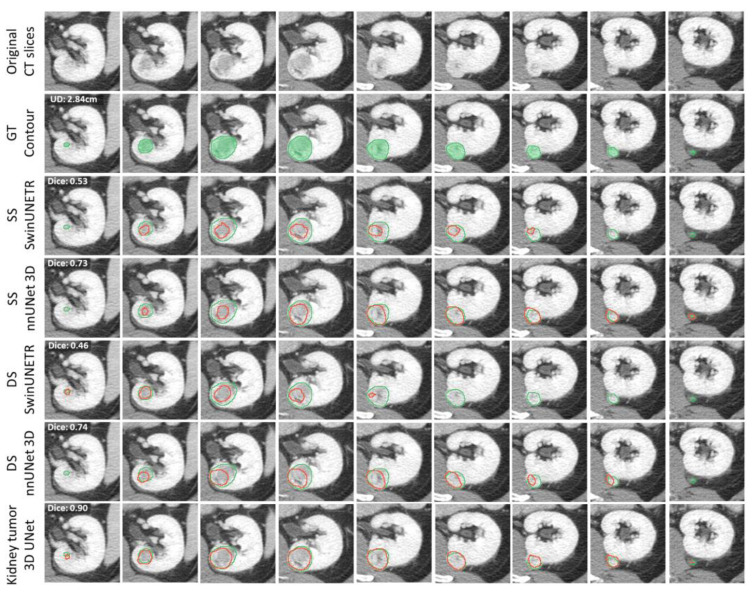
Examples of tumor segmentation results for small tumors: from left to right, the slices present the tumor in adjacent slices. The green contour indicates the GT, and the red contour indicates the resultant segmentation.

Figure S3 shows the kidney segmentation results, demonstrating the model’s accuracy. Figure 4 shows segmentation results for a small ccRCC tumor. The original CECT image slices display a tumor with a unidirectional diameter (UD) of 2.84 cm, shown across adjacent slices for comparison. The green contour represents the GT, while the red contour represents the segmentation produced by each AI model. The third and fourth rows show segmentation results from the SS models, while the fifth and sixth rows show results from the DS models. Both the SS and DS SwinUNETR models exhibit under-segmentation. The final row features the proposed Kidney Tumor 3D UNet, which achieves the highest Dice score of 0.90. These results demonstrate varying levels of accuracy, with the Kidney Tumor 3D UNet providing the closest match to the GT and superior performance in segmenting small tumors, highlighting the effectiveness of its architectural enhancements.

### 3.3. Further Segmentation Analysis for Small Tumors (≤4 cm)

**Figure 5:**
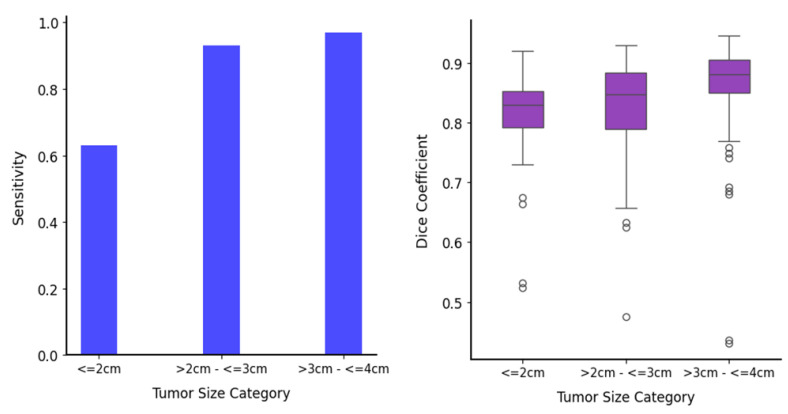
Detection and segmentation results for small tumor subcategories in private data: (**left**) sensitivity and (**right**) Dice coefficient across different tumor size categories for the proposed Kidney Tumor 3D UNet model.

To highlight the advantages of the proposed Kidney Tumor 3D UNet model for small tumors, out of a total of 607 tumors in the private data, we further analyzed its performance across the following three subcategories: ≤2 cm (44 tumors), >2 cm–≤3 cm (97 tumors), and >3 cm–≤4 cm (110 tumors). This analysis focused on the homogeneous ccRCC subtype to provide insights into the model’s effectiveness and robustness compared to other DS models. Figure 5 shows the detection and segmentation results for these subcategories, revealing that sensitivity for tumors with a UD ≤ 2 cm was lower than for the larger subcategories, likely due to unreliable CECT attenuation in smaller tumors [45].

The sensitivity analysis reveals that the proposed model consistently achieves higher sensitivity across these size subcategories. For tumors ≤ 2 cm, sensitivity values are 0.50 for DS_SwinUNETR, 0.54 for DS_nnUNet-3D, and 0.63 for the Kidney Tumor 3D UNet, representing a 26% improvement over DS_SwinUNETR and a 16.7% improvement over DS_nnUNet-3D. For tumors > 2 cm–≤3 cm, sensitivity values are 0.81 for DS_SwinUNETR, 0.83 for DS_nnUNet-3D, and 0.93 for the Kidney Tumor 3D UNet, indicating a 14.8% increase over DS_SwinUNETR and a 12% increase over DS_nnUNet-3D. For the >3 cm–≤4 cm subcategory, sensitivity values are 0.93, 0.96, and 0.97, respectively. These results highlight the Kidney Tumor 3D UNet’s ability to correctly identify tumors, particularly smaller ones.

In terms of Dice scores, the proposed model shows better performance. For tumors ≤ 2 cm, average Dice values are 0.77 for DS_SwinUNETR, 0.74 for DS_nnUNet-3D, and 0.81 for the Kidney Tumor 3D UNet, reflecting a 5.2% improvement over DS_SwinUNETR and a 9.5% improvement over DS_nnUNet-3D. For the >2 cm–≤3 cm category, average Dice values are 0.79 for both DS models and 0.83 for the Kidney Tumor 3D UNet, showing a 5.1% increase. In the > 3cm–≤4 cm category, average Dice values are 0.82, 0.83, and 0.86 respectively, representing a 4.9% improvement. The consistently higher Dice scores indicate better overlap with the GT and more accurate segmentation. These analyses confirm that the Kidney Tumor 3D UNet model excels in detecting and segmenting small tumors and maintains high performance across all subcategories.

## 4. Discussion

In this study, we developed a fully automated detection and segmentation algorithm for kidneys and kidney tumors in CECT images, utilizing transformer and 3D UNet architectures. Detecting kidney tumors, especially those ≤ 4 cm, is a crucial yet tedious task in clinical practice. Although various UNet architectures have been extensively studied for kidney tumor segmentation [29,31,32,33], our experiments revealed that these models often miss small tumors due to insufficient feature extraction and exhibit high FPs due to expansive search areas. To address these issues, we employed a DS approach and incorporated residual connections and attention mechanisms to enhance feature extraction. Our proposed AI model demonstrated accurate detection and segmentation of RCC tumors and the kidney organ across two different datasets. Notably, we trained our model using the publicly available KiTS23 dataset and tested it on a large external validation dataset consisting of 592 CECT scans from our institute, which includes a well-designed patient cohort with the ccRCC subtype acquired using different CECT systems.

Our results demonstrate that the DS approach effectively reduces FPs and increases the detection rate by narrowing the search space to relevant areas and excluding other organs. SOTA transformer models such as SwinUNETR and UNETR excel in detection and segmentation tasks, especially when the object size is significant relative to the search space. This success is also attributed to the use of homogeneous objects, large training datasets, and pretrained weights, which enhance the models’ ability to generalize and accurately segment target structures. This justified our use of SwinUNETR for kidney organ segmentation. Conversely, CNN-based models tend to perform poorly in large search spaces but excel with a reduced search space, which is particularly relevant in the complex domain of medical imaging. CNNs are adept at identifying local features, while ViTs may lose reasoning ability when the feature space is too localized.

Existing DS segmentation models often overlook the importance of the initial stage, treating it as merely a coarse step. However, our research indicates that this oversight can increase FPs due to poor organ localization, especially with large and heterogeneous datasets common in clinical practice. Thus, the initial localization process is as crucial as the tumor detection and segmentation process. We found that enhancing localization is essential for the success of the second stage. In our approach, we expanded the VOI by 25% in the first stage, ensuring that the tumor detection and segmentation network had access to sufficient background features, striking a balance between too much and too little contextual information.

**Figure 6:**
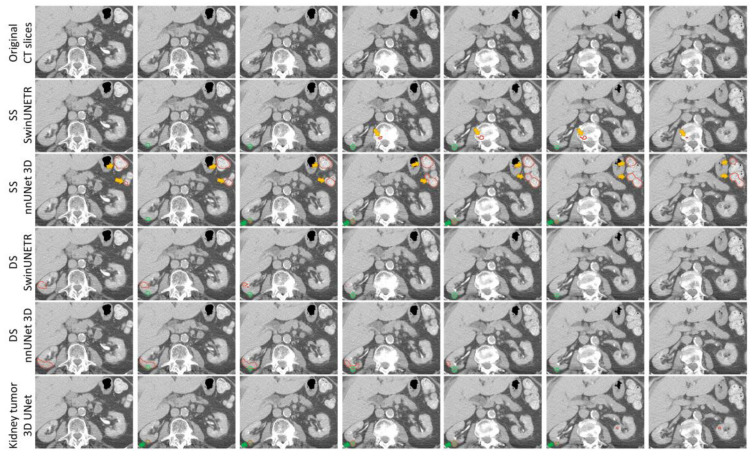
Comparison of false positives across all models. The green contour represents the GT, while the red contour represents the AI segmentation result. Yellow arrows indicate FPs outside the kidney region, and green arrows show correctly identified tumors.

Figure 6 compares segmentation results across all models, illustrating their performance in terms of FPs. The original CECT image slices display a tumor, shown across adjacent slices for comparison. The SS models show FPs outside the kidney region, as indicated by the yellow arrows. As shown in Figure 6, SS SwinUNETR misclassifies the kidney tumor as being in the vertebral body area, while SS nnUNet 3D mistakenly identifies a tumor in the small intestine, which is outside the kidney, highlighting the disadvantage of SS models in predicting outside the VOI. The Kidney Tumor 3D UNet outperforms all benchmark models in both sensitivity and FPC.

We observed that the Dice scores for small and medium-sized tumors in the private data were notably better than those in KiTS23 (Figure 3). This discrepancy likely results from the clearer definition and visibility of ccRCC tumors, which are more homogeneous in the private data (Figure S5) compared to the heterogeneous tumor subtypes in KiTS23. To further investigate, we compared the ccRCC cases across both datasets. The resulting boxplots (Figure S4) show quite similar distributions and medians, suggesting that the ccRCC subtype shows consistent segmentation performance across the two datasets. Additionally, our analysis of non-ccRCC subtype Dice scores (Figure S6) revealed that the ‘other’ subtype exhibited the lowest performance. These findings highlight the challenges of achieving consistent segmentation results across diverse tumor subtypes, emphasizing the need for careful consideration of subtype variability in model evaluation.

Additionally, we observed that as tumor size increases, the advantage of using DS models over SS models becomes less pronounced in terms of Dice score. For large tumors, SS_SwinUNETR and SS_nnUNet-3D achieved Dice scores of 0.84 in KiTS23, with slight improvements in the private data, reaching 0.90 for SS_SwinUNETR and 0.88 for SS_nnUNet-3D. DS models DS_SwinUNETR and DS_nnUNet-3D achieved Dice scores of 0.86 and 0.90, respectively, in the private data. However, FPC values reveal further nuances in model performance. SS_nnUNet-3D, despite its high Dice score, exhibited higher FPC values of 0.19 in KiTS23 and 0.26 in the private data, indicating a greater number of false positives compared to other models. On the other hand, SS_SwinUNETR showed lower FPC values, with 0.09 in KiTS23 and 0.05 in the private data. In contrast, DS models showed consistently lower FPC values, with DS_SwinUNETR recording 0.05 in KiTS23 and 0.02 in the Private data, and DS_nnUNet-3D recording 0.10 in KiTS23 and 0.03 in the private data. These results suggest that for large tumors, while DS models offer slight improvements in Dice scores, their more significant benefit lies in reducing false positives, thereby providing more reliable segmentation.

Despite achieving high performance in RCC tumor detection and segmentation, our research has several limitations. First, the private dataset only includes annotations for malignant tumors, without labels for benign tumors or cysts. To ensure consistency in the training process, we aligned the KiTS23 dataset by focusing on malignant tumor labels and not considering benign tumors and cysts, despite KiTS23 having labels for kidney, tumor, and mass. This resulted in a few FP cases due to cyst detection. Mitigating this issue would require annotating cysts in our private dataset or incorporating a portion of it into the training process. However, we did not use the private dataset for training in this research to evaluate the model’s adaptability to unseen data. Secondly, the private test data included only the ccRCC subtype, which limits our ability to generalize the model’s performance to datasets with multiple RCC subtypes. However, the training and validation data did include other RCC subtypes, although ccRCC was the predominant focus. Future work will expand experiments to include a larger dataset with a more diverse range of RCC subtypes.

We believe that ViTs are gaining popularity due to their capabilities, despite requiring more data and computational resources for training. As SOTA ViTs are combined with CNNs to create hybrid models, further experimentation is needed to understand how to effectively integrate the feature extraction strengths of CNNs with the global contextual understanding of ViTs. Our future work will focus on developing hybrid models by optimally fusing CNN layers with ViTs to leverage their combined power. Additionally, we aim to create a single recursive network that achieves optimal results for both organ and tumor segmentation, simplifying the training and execution phases and making the framework more efficient and streamlined.

## 5. Conclusions

In conclusion, we have presented a fully automated, end-to-end model for kidney organ and tumor detection and segmentation in CECT images. Our model has demonstrated the capability to accurately detect and segment kidney tumors of various sizes, achieving performance comparable to that of human experts and exhibiting significant robustness on unseen data. This highlights the potential of our deep learning model for integration into clinical workflows, providing a valuable tool to enhance diagnostic precision in radiology.

## Figures and Tables

**Table 1:** Segmentation performance for kidney organ in KiTS23.

Model	Dice	Precision	*p*-Value
nnUNet-3D	0.95 ± 0.05	0.95 ± 0.08	0.0498
SwinUNETR	0.97 ± 0.02	0.97 ± 0.02	-

**Table 2:** Tumor detection performance for kidney organ for the KiTS23 and private datasets.

Model\Metric	Small Tumors				Medium Tumors				Large Tumors			
	KiTS23		Private		KiTS23		Private		KiTS23		Private	
	Sen	FPC	Sen	FPC	Sen	FPC	Sen	FPC	Sen	FPC	Sen	FPC
SS_SwinUNETR	0.62	2.04	0.70	1.87	0.96	0.20	0.95	0.19	0.87	0.09	0.96	0.05
SS_nnUNet-3D	0.59	2.71	0.62	2.81	1.00	0.75	0.98	0.69	0.91	0.19	0.99	0.26
DS_SwinUNETR	0.67	0.91	0.80	0.60	1.00	0.24	0.99	0.12	0.91	0.05	1.00	0.02
DS_nnUNet-3D	0.74	0.60	0.84	0.33	1.00	0.23	0.98	0.08	0.96	0.10	0.99	0.03
Proposed Method	0.90	0.23	0.89	0.15	1.00	0.08	0.99	0.03	0.96	0.04	1.00	0.01

## Data Availability

The KiTS23 dataset is publicly available at https://github.com/neheller/kits23 (accessed on 4 April 2024).

## Supplementary Materials

The following supporting information can be downloaded at: https://www.mdpi.com/article/10.3390/tomography11010003/s1, Figure S1: Sensitivity bar chart comparing all models across the KiTS23 and private data.; Figure S2: FPC for all the models across the KiTS23 and private data.; Figure S3: Kidney segmentation results: The first column shows the original CT slices, the second column displays the Ground Truth annotations, the third column presents the results from the nnUNet 3D model, and the fourth column shows the results from the SwinUNETR model. Selected slices are presented to represent segmentation performance across the region of interest.; Figure S4: Boxplot of Dice scores only for ccRCC subtype, with statistical significance for all models across different tumor sizes in KiTS23 and private data. The statistical significance is indicated as follows: **** (*p* ≤ 0.0001), *** (*p* ≤ 0.001), ** (*p* ≤ 0.01), * (*p* ≤ 0.05).; Figure S5: Distribution of KiTS23 validation set tumor histological subtypes by category.; Figure S6: Distribution of Dice scores of KiTS23 validation set by tumor histological subtypes.; Table S1: Segmentation Results for small-sized kidney tumors KiTS23 and private data.; Table S2: Segmentation Results for medium-sized kidney tumors KiTS23 and private data.; Table S3: Segmentation Results for large-sized kidney tumors KiTS23 and private data.
