# Supplementary material for: Dual-Stage AI Model for Enhanced CT Imaging: Precision Segmentation of Kidney and Tumors
Source: Tomography. 2025 Jan 3;11(1):3. doi: 10.3390/tomography11010003 (PMC11769543; doi:10.3390/tomography11010003)
Supplement: Supplementary file 1 [file tomography-11-00003-s001.zip › tomography-3328991-supplementary.pdf]

Supplementary Materials

Table S1. Segmentation Results for small-sized kidney tumors KiTS23 and private data.

Metrics / Models	Dataset	Dice	Precision	VD	OSR	USR
SS_SwinUNETR	KiTS23	0.80 ± 0.14	0.88 ± 0.10	0.13 ± 0.4	0.13 ± 0.13	0.22 ± 0.20
	Private	0.79 ± 0.12	0.80 ± 0.12	0.14 ± 0.13	0.26 ± 0.29	0.16 ± 0.19
SS_nnUNet-3D	KiTS23	0.76 ± 0.14	0.85 ± 0.15	0.16 ± 0.16	0.18 ± 0.28	0.26 ± 0.20
	Private	0.77 ± 0.13	0.79 ± 0.14	0.14 ± 0.13	0.29 ± 0.39	0.19 ± .018
DS_SwinUNETR	KiTS23	0.81 ± 0.09	0.86 ± 0.13	0.13 ± 0.10	0.17 ± 0.20	0.20 ± 0.15
	Private	0.80 ± 0.12	0.85 ± 0.12	0.13 ± 0.13	0.19 ± 0.26	0.19 ± 0.18
DS_nnUNet-3D	KiTS23	0.80 ± 0.12	0.83 ± 0.12	0.12 ± 0.11	0.21 ± 0.24	0.18 ± 0.17
	Private	0.80 ± 0.10	0.77 ± 0.12	0.14 ± 0.11	0.31 ± 0.25	0.13 ± 0.15
Kidney tumor 3D UNet	KiTS23	0.84 ± 0.11	0.90 ± 0.11	0.10 ± 0.12	0.12 ± 0.22	0.18 ± 0.15
	Private	0.84 ± 0.08	0.82 ± 0.09	0.11 ± 0.09	0.23 ± 0.16	0.10 ± 0.14

Table S2. Segmentation Results for medium-sized kidney tumors KiTS23 and private data.

Metrics / Models	Dataset	Dice	Precision	VD	OSR	USR
SS_SwinUNETR	KiTS23	0.85 ± 0.12	0.89 ± 0.08	0.08 ± 0.10	0.11 ± 0.09	0.16 ± 0.16
	Private	0.86 ± 0.09	0.85 ± 0.09	0.09 ± 0.08	0.18 ± 0.18	0.10 ± 0.13
SS_nnUNet-3D	KiTS23	0.83 ± 0.11	0.85 ± 0.15	0.10 ± 0.09	0.20 ± 0.30	0.16 ± 0.12
	Private	0.85 ± 0.10	0.85 ± 0.11	0.08 ± 0.09	0.18 ± 0.24	0.12 ± 0.13
DS_SwinUNETR	KiTS23	0.87 ± 0.07	0.91 ± 0.08	0.07 ± 0.07	0.10 ± 0.10	0.15 ± 0.12
	Private	0.87 ± 0.10	0.87 ± 0.10	0.08 ± 0.10	0.16 ± 0.23	0.12 ± 0.13
DS_nnUNet-3D	KiTS23	0.86 ± 0.11	0.84 ± 0.17	0.08 ± 0.11	0.25 ± 0.43	0.10 ± 0.07
	Private	0.87 ± 0.08	0.85 ± 0.10	0.08 ± 0.08	0.19 ± 0.22	0.09 ± 0.11
Kidney tumor 3D UNet	KiTS23	0.89 ± 0.07	0.90 ± 0.08	0.06 ± 0.07	0.11 ± 0.10	0.09 ± 0.11
	Private	0.89 ± 0.08	0.86 ± 0.09	0.07 ± 0.08	0.17 ± 0.20	0.07 ± 0.10

Table S3. Segmentation Results for large-sized kidney tumors KiTS23 and private data.

Metrics / Models	Dataset	Dice	Precision	VD	OSR	USR
SS_SwinUNETR	KiTS23	0.84 ± 0.14	0.93 ± 0.05	0.11 ± 0.13	0.06 ± 0.05	0.20 ± 0.20
	Private	0.90 ± 0.08	0.89 ± 0.08	0.06 ± 0.07	0.14 ± 0.25	0.07 ± 0.10
SS_nnUNet-3D	KiTS23	0.84 ± 0.11	0.92 ± 0.07	0.10 ± 0.10	0.07 ± 0.08	0.20 ± 0.15
	Private	0.88 ± 0.09	0.90 ± 0.08	0.06 ± 0.07	0.12 ± 0.20	0.12 ± 0.12
DS_SwinUNETR	KiTS23	0.86 ± 0.10	0.93 ± 0.06	0.08 ± 0.11	0.07 ± 0.07	0.17 ± 0.15
	Private	0.90 ± 0.07	0.91 ± 0.06	0.06 ± 0.07	0.10 ± 0.10	0.09 ± 0.12
DS_nnUNet-3D	KiTS23	0.86 ± 0.11	0.87 ± 0.15	0.10 ± 0.11	0.19 ± 0.34	0.12 ± 0.12
	Private	0.90 ± 0.07	0.89 ± 0.10	0.05 ± 0.07	0.14 ± 0.22	0.07 ± 0.07
Kidney tumor 3D UNet	KiTS23	0.90 ± 0.06	0.93 ± 0.07	0.06 ± 0.05	0.07 ± 0.08	0.11 ± 0.09
	Private	0.92 ± 0.06	0.90 ± 0.08	0.04 ± 0.06	0.12 ± 0.24	0.05 ± 0.06

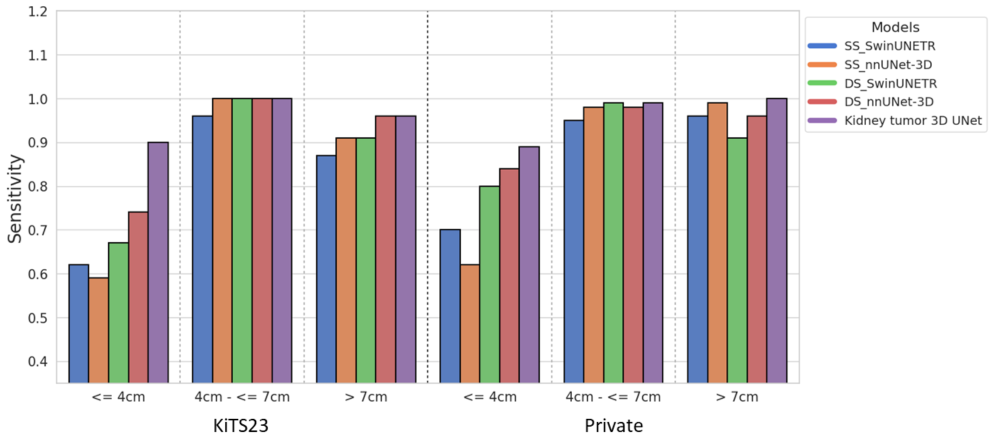

Figure S1. Sensitivity bar chart comparing all models across the KiTS23 and private data.

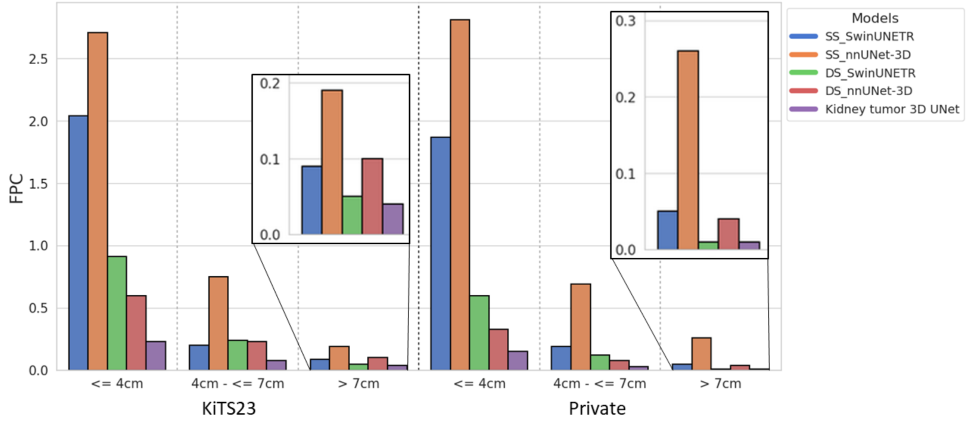

Figure S2. FPC for all the models across the KiTS23 and private data.

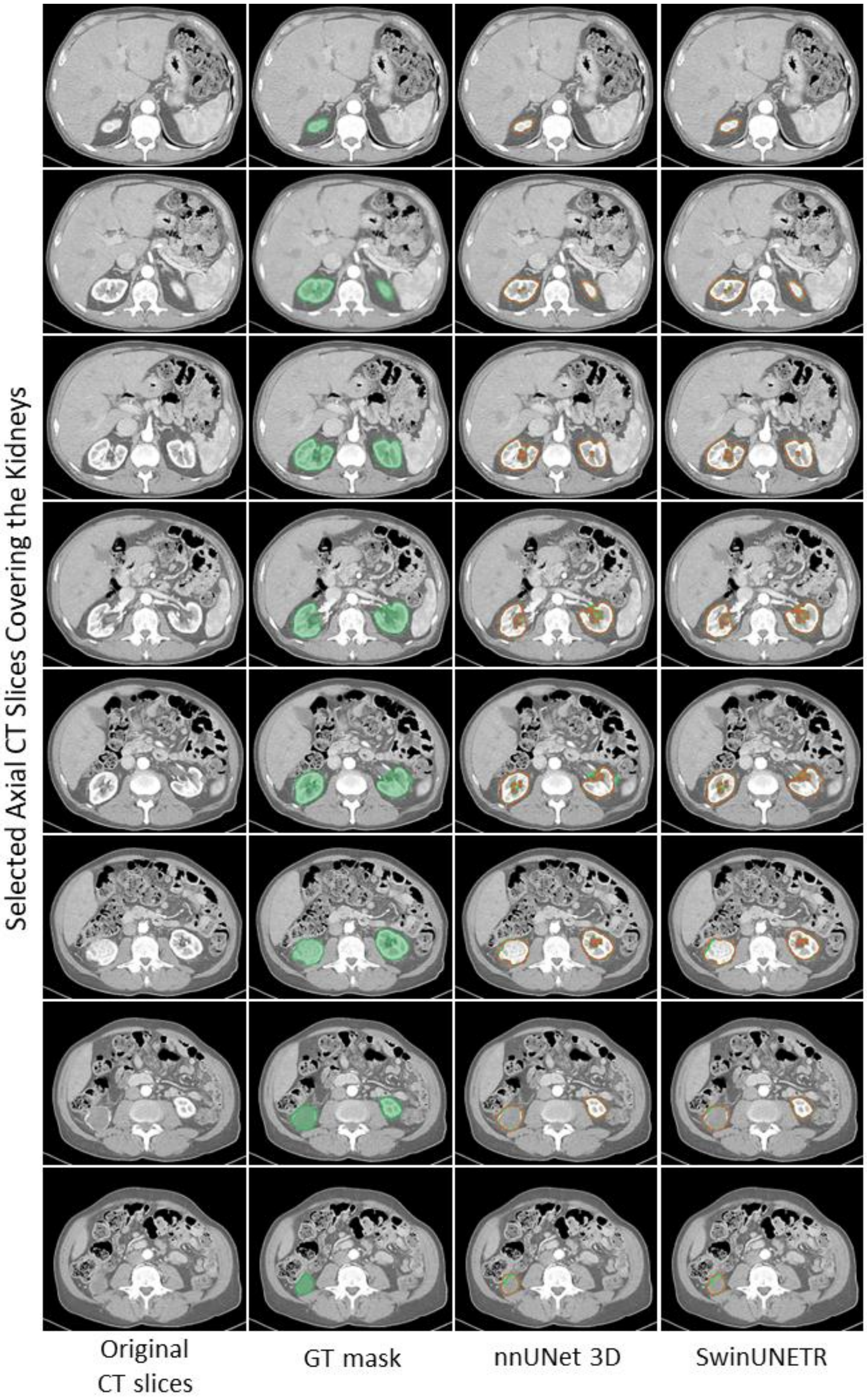

Figure S3. Kidney segmentation results: The first column shows the original CT slices, the second column displays the Ground Truth annotations, the third column presents the results from the nnUNet 3D model, and the fourth column shows the results from the SwinUNETR

model. Selected slices are presented to represent segmentation performance across the region of interest.

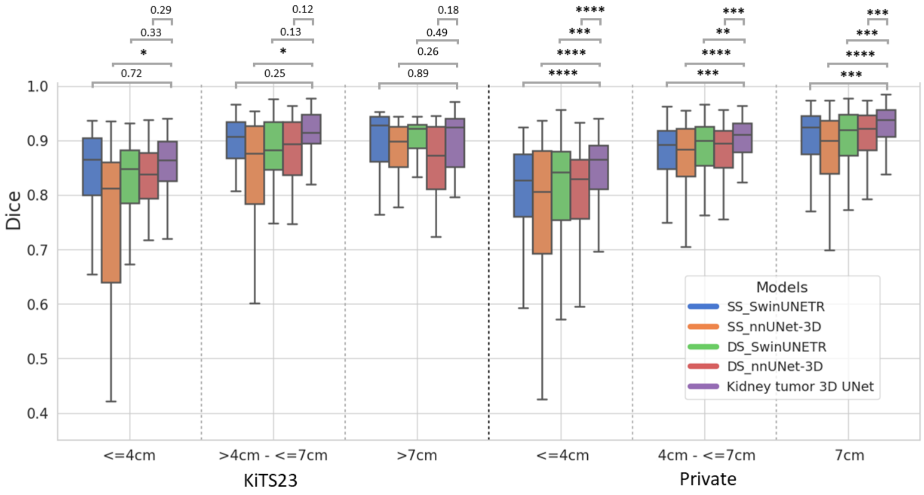

Figure S4. Boxplot of Dice scores only for ccRCC subtype, with statistical significance for all models across different tumor sizes in KiTS23 and private data. The statistical significance is indicated as follows: **** ($p \leq 0.0001$), *** ($p \leq 0.001$), ** ($p \leq 0.01$), * ($p \leq 0.05$).

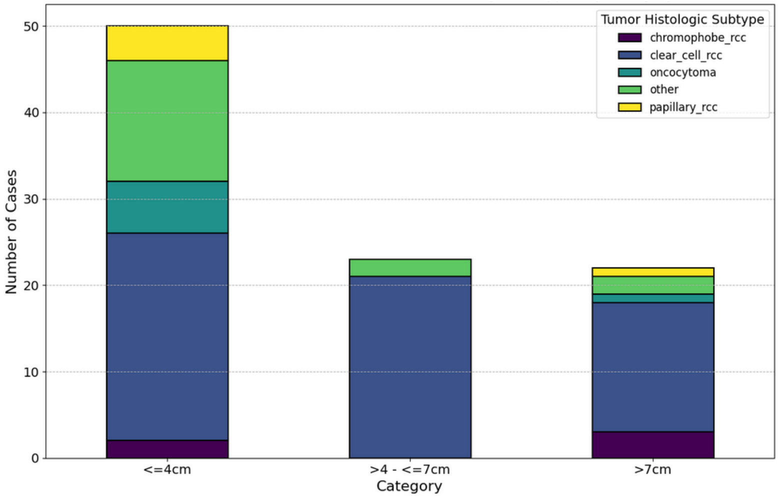

Figure S5. Distribution of KiTS23 validation set tumor histological subtypes by category.

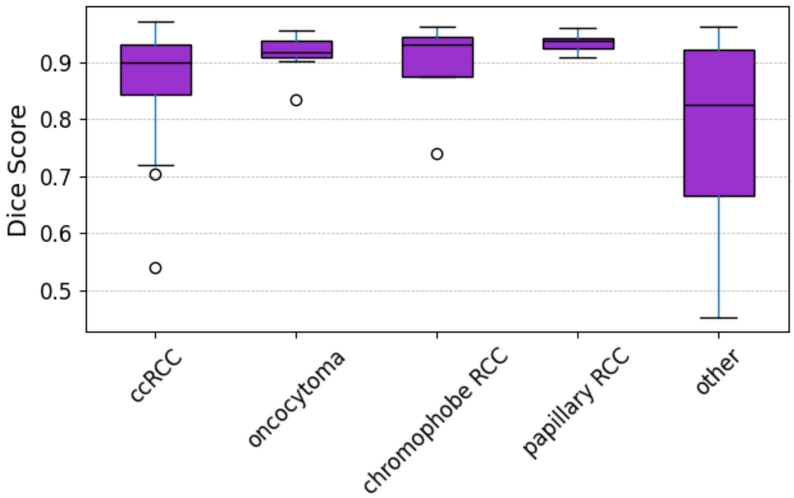

Figure S6. Distribution of Dice scores of KiTS23 validation set by tumor histological subtypes.
